# Advances in ME/CFS: Past, Present, and Future

**DOI:** 10.3389/fped.2019.00131

**Published:** 2019-04-18

**Authors:** Kenneth J. Friedman

**Affiliations:** Retired, Plantation, FL, United States

**Keywords:** ME/CFS, HIV/AIDS, HISTORY, comparison, patient care, research

## Abstract

The forerunner of what is today termed myalgic encephalomyelitis/chronic fatigue syndrome (ME/CFS) was described by the U.S. Public Health Service in 1934. At the present time, we still do not know its cause and/or how to detect it by routine clinical laboratory tests. In consequence, the pathological nature of ME/CFS has been overlooked and the disease has been stigmatized by being mislabeled as psychosomatic or somatoform illness. Such misperceptions of the disease have led to insufficient research exploration of the disease and minimal to absent patient care. A 2015 Institute of Medicine report on the illness declared ME/CFS a disease affecting up to 2.5 million Americans and chastised the U.S. government for doing little to research the disease and to support its patients. Clinicians who currently treat this disease declare it to be more devastating than HIV/AIDS. A comparison of the histories of the two diseases, an examination of the current status of the two diseases, and a listing of the accomplishments that would be needed for ME/CFS to achieve the same level of treatment and care as currently experienced by patients with HIV/AIDS is provided.

## Introduction

Myalgic Encephalomyelitis/Chronic Fatigue Syndrome (ME/CFS) is a stigmatized, multisystem, complex, chronic disease that potentially affects up to 2.5 million Americans (1). Initially described in the United States in 1934 (2), it has alternatively been mischaracterized as hysteria (3), psychosomatic (4), and psychological illness (5).

The characterization of ME/CFS as a psychosomatic illness has led to a proliferation of literature describing cognitive behavioral therapy (CBT) and graded exercise therapy (GET) as non-physiological approaches to treat the illness. Patients have claimed harm from these therapies, but the pro-CBT literature alleges otherwise. Recently, literature disputing the benefits of CBT and GET has emerged (6), and the decision has been reached to withdraw some of the CBT and GET studies (7).

Many members of the healthcare community and the public are dismissive of ME/CFS as a disease (8) and, therefore, according to criteria published by the U.S. Institute of Medicine (now the National Academy of Medicine), ME/CFS qualifies as a stigmatized illness (9). Such illnesses are difficult to treat because of healthcare provider dismissive attitudes (10) and the lack of community support for patients (11). Compounding these difficulties for ME/CFS patients is the failure to find consistent abnormalities in routine, clinical laboratory tests which can confirm a ME/CFS diagnosis. Without the availability of an accepted, laboratory, diagnostic test indicating the presence of a unique infectious agent, metabolic or organ system abnormality, there is reluctance to believe that ME/CFS is a pathophysiological illness. In consequence, the search for the etiological agent or agents of ME/CFS, specific treatments for its relief, medications or a vaccine specific for the disease have been hampered. Without such knowledge and tools, physicians and other healthcare providers receive little healthcare provider education about the disease. Knowing the previous struggles for research and treatment, and the current status of patient care for a disease which similarly suffered from stigmatization, unknown disease etiology, and no known treatment, but has progressed much further in a shorter amount of time, helps clarify what can be achieved for ME/CFS in the future.

Our current understanding of ME/CFS is that it is a complex, chronic, debilitating, physical disease characterized by post-exertional malaise, severe, and debilitating fatigue, cognitive problems, sleep dysfunction, pain, and immune, autonomic, neurological, endocrine, and gastrointestinal symptoms (12). The severity of symptoms varies from day-to-day within the patient, and varies among patients. Severity of disease is graded from patients being mildly affected, through moderately affected, to the severely affected. The severely affected are either house- or bedbound, may be unable to move, speak or tolerate light (13). Post-exertional malaise (PEM) is considered one of the key symptoms of ME/CFS and is defined as the exacerbation of the patient's symptoms following minimal physical or mental activity, occurring hours, days, or weeks after the triggering activity, and lasting for disproportionately long lengths of time (days, weeks, or months).

Diagnosis of ME/CFS is difficult. Without the availability of a diagnostic test, several sets of diagnostic criteria have been proposed. Fukuda et al. (14) has been the most popular, but it must be remembered that it was proposed as, and is, a research case definition never intended for clinical diagnosis. The recent Institute of Medicine report (1) proposes simplified criteria which have not been widely embraced. Without a disease specific test or biomarker, there is no choice but to base diagnosis on the symptoms reported by the patient. Some symptoms are considered mandatory for the diagnosis, whereas others are considered supportive. There is a reluctance to diagnose adults with ME/CFS until they have been ill for at least 6 months and their co-morbidities have been addressed. For children and adolescents, and for humanitarian reasons, the length of time of required illness prior to receiving a ME/CFS diagnosis has been shortened to 3 months (15), but the treating physician should use his/her own discretion.

Treatment of ME/CFS has been difficult. The availability of treatment and therapeutic approach varies among countries. Some countries lack awareness of ME/CFS (16), others subscribe to ME/CFS as treatable by changing the way the patient thinks and behaves through a CBT approach (17, 18), while some have embraced ME/CFS being an organic disease in practice (19) but fail to reach all patients and caregivers in need (20). Recently, O'Leary (21) has put forward the argument that despite a previous, long-term, professional consensus that CFS be classified as a psychosomatic illness, the insistence of globally respected health authorities that ME/CFS be treated as a serious, biological disease (1) raises ethical concerns as to whether efforts to continue treating ME/CFS as a mental disorder (in the U.K.) should continue.

While HIV/AIDS was far more devastating than ME/CFS when what was to be called AIDS was first reported, it no longer is. At this point in time it is comparable or less severe than ME/CFS for the majority of patients as witnessed by the following two quotes from physicians who treat both: (1) “In my experience, *(ME/CFS) is one of the most disabling diseases that I care for, far exceeding HIV disease except for the terminal stages.”*—Dr. Daniel Peterson (22). (2) *I split my clinical time between the two illnesses, and I can tell you if I had to choose between the two illnesses (in 2009) I would rather have H.I.V. But C.F.S., which impacts a million people in the United States alone, has had a small fraction of the research dollars directed toward it.”—*Dr. Nancy Klimas, AIDS and CFS researcher and clinician, University of Miami (23).

## The Past

It has been over 80 years since the first literature-documented outbreak of ME/CFS in the United States in 1934 (2). Yet, this illness remains poorly characterized and there is no defined course of treatment. In contrast, the time span from the first description of HIV/AIDS to the development of treatment with Federal Drug Administration (FDA)-approved drugs was less than a decade (24). Attempts to classify ME/CFS have been misleading. The majority of ME/CFS cases have been classified as, “sporadic,” despite the infectious origin of most cases: Hickie et al. (25) report that 6 % of Epstein Barr Virus, Ross River Virus and Q fever patients develop ME/CFS, with the severity of viral infection being the best predictor of contracting ME/CFS. In 1955, McEvedy and Beard (3) labeled a cluster outbreak of the illness as “mass hysteria,” and the *New York Times* cheekily renamed the illness, “Yuppie Flu,” mischaracterizing the illness as exclusively affecting young and upcoming professionals (26). To the contrary, research shows that ME/CFS affects all socioeconomic groups of the American population, with socioeconomically disadvantaged populations perhaps being more greatly affected (27).

Mischaracterization of ME/CFS has led to inappropriate and, for some, harmful treatment options (28). Characterization of ME/CFS as a psychosomatic illness has led to the belief that cognitive behavioral therapy (CBT) and graded exercise therapy (GET) are therapeutic if not curative (6). The publications demonstrating the therapeutic and potentially curative values of CBT and GET have now been challenged (18). Attempts to correct the literature are currently underway (29). When a child has ME/CFS, the parent or parents may be accused of Munchausen's Syndrome By Proxy (MSBP). In some cases, these children have been removed from the home leading to increased severity of illness (30).

Some of the professional scientists who have pursued ME/CFS have jeopardized their careers and livelihood (31). Medical school clinicians have been told to stop seeing ME/CFS patients because they require too much of a clinician's time, and, if they do not stop, they will need to work elsewhere. Researchers have been told that ME/CFS research will not be considered for their promotions, and if they are not promoted, they will need to leave the institution. Medical educators have been told that ME/CFS educational activities are not, “professional,” and those activities are banned from the workplace. While the NIH State of Knowledge ME/CFS Workshop, held in 2011, exposed and documented these problems (31), there has been no documentation of NIH objecting to any of these practices. NIH could withhold or limit funding to institutions which discourage or do not permit medical research and patient care for diseases that are of importance to the NIH. Not even the American Association of University Professors (AAUP), which claims academic freedom as its core mission (32), has objected to academic, institutional bans on ME/CFS research, clinical care, and educational activities.

*The Guardian* summarized the history of inquiry into ME/CFS this way: “*… for much of the past three decades, CFS has been treated as the proverbial skeleton in the closet of the medical world. Potential researchers have been scared off by the stigma associated with the disease, and government funding has been non-existent”. “When I was a medical student in the ‘90s, we were instructed that CFS patients could not be seen in our clinic,” Montoya recalls. “And a letter was sent out to those patients telling them not to come”* (33).

The U.S. government's support of ME/CFS research and patient care has been less than stated and not commensurate with the burden of the disease. When the U.S. Congress allocated funds for a Centers for Disease Control and Prevention (CDC) investigation of ME/CFS, the Director of the Chronic Fatigue Syndrome (CFS) Program (CFS is the former name of ME/CFS), Dr. William C. Reeves, filed a whistleblower complaint against the agency, alleging that millions of dollars committed to CFS research had actually been spent on other activities (34). But, it took a request from Sen. Harry Reid, D-NV to the General Accounting Office (GAO) to obtain a report (35). The GAO report stated that about one-third of the funds had been spent on non-CFS-related activities (36). Publicly exposed, the CDC agreed to restore the misappropriated funds and to institute measures which would prevent recurrences (37). According to the U.S. Government General Accounting Office (38): “ *at CDC, the lengthy and uncertain process for allocating CFS funds to the branch responsible for most of the CFS work has resulted in delays in undertaking particular projects; … further, CDC's redirection of funds has resulted in reductions in CFS resources that have impeded the agency's CFS research…coordination between CDC and NIH and their use of input from external researchers and patient advocates in developing agency research programs have been limited.”*

From the mid-1990's until this year, there have been signs of increased interest of the federal government in ME/CFS: In 1997, the Chronic Fatigue Syndrome Coordinating Committee, composed of researchers, clinicians and patient advocates, was formed and tasked with advising the U.S. Secretary of Health and Human Services on matters related to ME/CFS (39). In 2003, the Coordinating Committee was replaced by the Chronic Fatigue Syndrome Advisory Committee (CFSAC) (40). In 2006, then Director of the CDC Julie Gerberding launched a, “Spark Awareness,” campaign for ME/CFS which included a ME/CFS public awareness campaign, and the distribution of a *CFS Toolkit for Health Care Professionals* to aid in the diagnosis and treatment of ME/CFS (41). However, despite those efforts, the recent studies of Tidmore et al. (42) and Sunnquist et al. (43) have shown that definitive care of persons with ME/CFS in the United States is still lacking.

In 2014, the Department of Health and Human Services issued a $1 million contract to the Institute of Medicine (IOM) for a report (44) on specific aspects of ME/CFS. The IOM Report (1) finds the federal response to ME/CFS deficient. In response to that report, both the National Institutes of Health (NIH) and the CDC promised to modify and enhance their ME/CFS programs, and to correct the deficiencies identified in the IOM report. A long-term plan or commitment which addresses all of the concerns raised in the IOM report has yet to be announced. Wadman (45) reported in *Science* magazine that NIH spending for ME/CFS would double, and NIH would solicit proposals for basic and clinical research centers. The CDC is revising and creating new educational materials for its ME/CFS web pages. The revised pages are pledged to reflect the content and recommendations of both the IOM report and the CDC's ME/CFS stakeholders' meeting held in September, 2016 (46).

While these are indications of improved attitudes toward ME/CFS in two key agencies of the U.S. federal government, there remain indications that the federal government has not fully embraced or accepted ME/CFS for the pathophysiological disease that it is: By its own admission, the NIH intramural, Clinical Center study of ME/CFS and an NIH-sponsored Pathways to Prevention meeting (47) were prompted by the 2015 IOM report. The recommendations of the Chronic Fatigue Syndrome Advisory Committee from its first research recommendation in 2004 to the date of publication of the IOM report were apparently insufficient to motivate the NIH to increase ME/CFS extramural funding or establish an intramural clinical program. An increase in research funding was attained subsequent to the IOM report (48). However, until 1999, NIH implied that ME/CFS is a women's disease (which it is not) by running its ME/CFS programs out of the Office of Research on Women's Health (49). A recent study finds 35 to 40 percent of adults diagnosed with ME/CFS are men (50) This fall, the Secretary of Health and Human Services decided to not renew the charter of the Chronic Fatigue Syndrome Advisory Committee stating the goals of the Advisory Committee had been achieved (51).

The U.S. government allocates far fewer research dollars for ME/CFS than it does for other chronic diseases of commensurate severity. For example, the estimated 2018 research expenditure for HIV/AIDS in the United States is $2.2 billion (52). With an estimated 1.1 million HIV/AIDS patients living in the U.S (53), the research expenditure per patient is $2,000. If the IOM report is correct, there may be more than double the number of ME/CFS patients living in the U.S. The ME/CFS research expenditure for FY 2017 was $13,967,704 (54), resulting in $5.58 spent per patient. The federal government spends 357 times more on HIV/AIDS research than on ME/CFS. The IOM report states, “*The committee was struck by the relative paucity of research on ME/CFS conducted to date…*” (1).

The federal government allocates more resources for orphan diseases—diseases that affect < 200,000 U.S. citizens—than it does for ME/CFS. The NIH orphan diseases program provides services which include a centralized database, liaison services, consortia funding and a contact registry (55). None of these services are provided for ME/CFS by any federal agency.

## The Present

ME/CFS is a worldwide disease. The reported prevalence of ME/CFS varies in different parts of the world. Son (56) searched prevalence studies in the literature and found prevalence studies from 13 countries. Within-country prevalence ranged from 0.0004 percent in Australia to 3.6 percent in the United States. Adolescent prevalence ranged from 0.9 percent in the United Kingdom to 0.11 in the Netherlands.

ME/CFS may affect up to 2.5 million Americans (1). An estimated 25 percent of ME/CFS patients have severe ME/CFS, i.e., are either house- or bed-bound at some point in their lives (57). ME/CFS patients need more care than patients with many other illnesses (1). Yet, there are regions in the United States where they do not receive any specialized, medical care (43). There is no laboratory test or diagnostic marker for ME/CFS (58). There is not one FDA-approved drug for the treatment of ME/CFS (59). In contrast, there are approximately 40 FDA approved drugs for the treatment of HIV/AIDS (60). And there is obviously no cure for ME/CFS (59).

Three recent documents, authored by the federal government itself, suggest the need for increased federal participation in the ME/CFS Agenda: (a) the Institute of Medicine Report (1), (b) the NIH Pathways to Prevention Report (61), and (c) the NIH State of Knowledge Workshop Report (62). In addition to documenting the discrimination experienced by ME/CFS patients, the clinicians who treat them, and the researchers who study their illness, the NIH State of Knowledge Workshop Report also identified a need for more interdisciplinary research, more researchers, and translational research, defined as starting at the bedside, going to the laboratory bench, and back to the patient again (63).

There are other reasons to increase federal support for ME/CFS research and patient care: (1) The federal government has an obligation to provide fair opportunity to all persons and eliminate impediments to fair opportunity: “*No persons should be denied social benefits on the basis of underserved disadvantageous properties*” (64). This concept of “fair opportunity” has led to calls to eliminate health disparities (65, 66).

(2) The federal government has an obligation to treat ME/CFS patients because they constitute a medically underserved population. ME/CFS patients qualify as a medically underserved population by virtue of geographic and financial barriers to care (42). Both Tidmore et al. (42) and Sunnquist et al. (43) found: (a) areas of the United States in which there are no ME/CFS specialized care providers, (b) areas in which there are an inadequate number of specialized care providers, (c) areas in which there are an inadequate number of knowledgeable primary care providers, and (d) a national lack of educational opportunities for physicians and medical students to obtain the didactic and clinical experience necessary to diagnose and to treat ME/CFS.

(3) The U.S. government has the ability to initiate a national, federal, healthcare program for an identified, disabled, patient population within the United States, a population that otherwise lacks equal access to healthcare and, therefore, represents a medically underserved population (67). These arguments were part of the justification for establishing multiple Centers of Excellence for ME/CFS in the United States, presented at the Chronic Fatigue Syndrome Advisory Committee meeting held May 17-18, 2016 and incorporated into recommendations submitted to the Office of the Secretary of Health of the United States (68). Currently, there is not one, federally sponsored or supported ME/CFS clinic in the United States.

The IOM report (1) held the promise of being a watershed moment for ME/CFS: A prestigious, independent, scientific body declared ME/CFS a disease, and the government response to it, inadequate. Thus, far, the responses of both the NIH and the CDC are not commensurate with the severity of the disease nor the number of people affected: Were the federal government to address the current needs of the ME/CFS community, it would need to provide ME/CFS funding commensurate to that of other diseases of similar severity that impact a similar number of patients (as, for example, HIV/AIDS). There would need to be commensurate numbers of treatment centers staffed by physicians capable of providing definitive care.

It has been 3 years since the publication of the 2015 IOM report. Were the IOM report to have had impact, there would be an increase in ME/CFS research, and an increase in public awareness of ME/CFS subsequent to the publication of the report. Such desired increases are not apparent in an examination of the number of research articles published per year in the scientific literature or in the number of articles published per year in the lay literature before and after the 2015 IOM report. These conclusions were reached by determining the number of ME/CFS citations per year in the scientific and lay literature before and after the IOM report. To ascertain the number of articles in the scientific literature, *PubMed* was queried searching article titles for any of the terms used as names for ME/CFS (myalgic encephalomyelitis, chronic fatigue syndrome, ME/CFS, CFS/ME, CFS, SEID, systemic exertion intolerance disease). Results are shown in Table 1.

**Table 1:** ME/CFS publications/year in the scientific literature.

**Year**	**# of ME/CFS articles published**
2010	204
2011	213
2012	153
2013	195
2014	158
2015	205
2016	197
2017	199
2018 to 12/1	178

To determine if the IOM report had impact on public awareness, the lay literature was similarly searched utilizing two databases: *Proquest U.S. Newstream* and *EBSCO's Academic Search Premier*. *Newstream* is described as a database covering newspapers, news websites, blogs, and many national and regional titles. *Academic Search* is described as an interdisciplinary database of newspapers, magazines and journals. The results of querying these two databases are shown in Table 2.

**Table 2:** ME/CFS publications/year in the lay literature.

**Year**	**Newstream articles/Year**	**EBSCO Premier articles/Year**
2010	328	252
2011	402	311
2012	253	227
2013	202	211
2014	257	171
2015	249	229
2016	182	191
2017	250	218
2018	166 (to 11/29/18)	141 (to 11/29/18

The hoped for increases in research publications and/or public awareness as expressed as increases in number of articles in the scientific or lay literature, subsequent to the publication of the IOM report, have not occurred 3 years after the publication of this report.

## The Future

The goals of ME/CFS researchers, healthcare providers, and patients are the same as the goals of others confronting different chronic, debilitating disease: the availability of palliative if not curative treatment, an understanding of the etiology of the disease, development of therapeutic agents specific for the disease, and the development of a vaccine or some other preventative measures. The IOM report (1) concludes that progress in these areas has been disappointingly slow. That opinion is supported by comparing the progress made in the research, treatment and prevention of ME/CFS to that of HIV/AIDS.

At the current time, a comparison between treatment and care of HIV/AIDS patients and ME/CFS patients is reasonable. Both are chronic conditions, with immunological underpinnings. However, whereas HIV (human immunodeficiency virus) has been identified as the cause of AIDS (69), several viruses (e.g., Epstein-Barr, Ross River, and Coxiella burnetti) are associated with the onset of ME/CFS (70). And there are reports of non-viral, physiological abnormalities which may trigger or contribute to the symptoms of ME/CFS (71). It is therefore possible that identifying the causative agent or agents for ME/CFS may take longer than the time needed to discover the AIDS virus. But whereas the HIV/AIDS virus was discovered within a decade, it has been more than eight decades since the description of ME/CFS, and the causative agent or agents have yet to be identified. In the United States, the latest CDC estimate of HIV/AIDS patients suggests that there may be twice as many ME/CFS patients [2.5 million estimated in the IOM report (1)] as HIV/AIDS patients [estimated to be 1.1 million patients by the (72)].

While HIV/AIDS was far more devastating than ME/CFS when the disease was first described, it no longer is. At this point in time it is comparable or less severe than ME/CFS for the majority of patients as attested to by Drs. Peterson and Klimas - two physicians who are well experienced in the treatment of both (22, 23).

At the onset of the AIDS epidemic, the diagnosis of AIDS was a death sentence (73). Today, a 20-year-old, infected with HIV, if appropriately treated, is expected to live into his/her 70's (74). AIDS advocacy (the AIDS Movement) was able to bring U.S. federal spending for AIDS to $3 billion/year, stimulate the development and distribution of 33 drugs in 7 seven different categories for the treatment of AIDS, and transform AIDS from a death sentence to a chronic, treatable condition (75). Many of those gains for HIV/AIDS patients continue today (76).

The accomplishments of the AIDS Community are enviable. From the first report of what would be named HIV/AIDS on June 5, 1981 (77):

- a Congressional Hearing on AIDS was held in < 1 year's time.
- $5 million was given to the CDC for surveillance and $10 million given to the NIH for research < 6 months later.
- A clinic dedicated to the treatment of AIDS patients opened approximately 1.5 years after the first reporting.
- The World Health Organization held its first AIDS meeting and began global surveillance for HIV/AIDS < 3 years after the first reporting.
- The cause of HIV/AIDS was announced by the National Cancer Institute 3 years after the first reporting.
- The first, specific therapeutic drug (antiretroviral agent) was approved 6 years after the first case report of AIDS.
- The Surgeon General mailed 107 million copies of a booklet, “Understanding AIDS,” to American households 7 years after the first case report.
- The Food and Drug Administration (FDA) permitted the importation of unapproved drugs for the treatment of HIV/AIDS 7 years after the first case report.
- The United Nations (UN) and World Health Organization (WHO) supported World AIDS Day 7 years after the first case report.
- Health Resources and Services Administration (HRSA) awarded HIV planning grants for, “systems of care,” 7 years after the first case report.

All this was accomplished by a living HIV/AIDS population in the United States of under 100,000 patients (78).

Despite HIV/AIDS currently being considered a manageable disease, the proposed U.S. HIV/AIDS budget for 2018 was $32 billion of which 85 % ($20.7 billion) was for domestic care and treatment programs, 9% ($3.1 billion) for domestic care and housing assistance, 7 % ($2.2 billion) for domestic research, and 2 % ($0.7 billion) for domestic prevention (52). There are no domestic care, treatment, prevention, or housing programs for ME/CFS patients. For ME/CFS, a disease estimated to have more than double the number of patients, and with a quality of life judged to be as or more diminished than that of HIV/AIDS, the disparity in patient care and patient benefits is unsettling.

AIDS patients were identified in 1981 and the virus causing AIDS was identified in 1984 (79). NIH expenditure for AIDS research for the period of 1981–1984 was $132,881,000 [calculated from Table 4.2, (80)]. Knowing that AIDS is caused by a single virus, whereas multiple viruses and other triggers precipitate ME/CFS, the research expenditure likely needed to determine the etiology of ME/CFS will be equal to or greater than the research expenditure required to determine the causal agent of AIDS. The federal government and the patient community need to be aware of the probable cost of identifying the causal agent or agents of ME/CFS.

The accomplishments for and by the HIV/AIDS Community, while attributed to HIV/AIDS “activism,” (81) make it clear that the U.S. government could have done and still can do much more for ME/CFS. The accomplishments for and by the HIV/AIDS Community may suggest goals for the ME/CFS Community. A lack of parity of research and benefits is logically and ethically difficult to justify.

With the non-renewal of the Chronic Fatigue Syndrome Advisory Committee charter by the U.S. Secretary of Health (82), there is no formal venue in which the inequalities of ME/CFS research and treatment of ME/CFS patients can be addressed. But, knowing what has not worked for the ME/CFS Community in the past, and knowing what has worked for others who have suffered similar disparities in disease-specific healthcare and medical research, may prove helpful to the severely underserved ME/CFS Community.

## Author's Note

“Advances in ME/CFS Research and Clinical Care: Past, Present and Future” is a Perspective article written to be the first article appearing in “Advances in ME/CFS Research and Clinical Care,” the invited, themed issue of Frontiers in Pediatrics of which I serve as Guest Editor. The purpose of the article is to provide the reader with background knowledge helpful for better appreciating the subsequent articles in the journal issue. Although what we currently call ME/CFS was described by the forerunner of the Centers for Disease Control and Prevention (in the United States) in the 1930's, it has been mischaracterized until 2015, when the Institute of Medicine declared the symptoms of the syndrome so severe, and the research supporting pathophysiological underpinnings of the syndrome sufficiently substantial, to declare the illness a disease.

The history of ME/CFS up to the publication of the IOM report is quite similar to the history of HIV/AIDS which was first identified in the early 1980's. However, the path or time to acceptance of HIV/AIDS as a “real” disease, and the development of drugs and treatment for it, have been far shorter.

A comparison of the two diseases is, therefore, inevitable, logical and useful.

## Author Contributions

The author confirms being the sole contributor of this work and has approved it for publication.

### Conflict of Interest Statement

The author declares that the research was conducted in the absence of any commercial or financial relationships that could be construed as a potential conflict of interest.
